# Psychological response of family members of patients hospitalised for influenza A/H1N1 in Oaxaca, Mexico

**DOI:** 10.1186/1471-244X-10-104

**Published:** 2010-12-03

**Authors:** Jesús Elizarrarás-Rivas, Jaime E Vargas-Mendoza, Maurilio Mayoral-García, Cuauhtémoc Matadamas-Zarate, Anaid Elizarrarás-Cruz, Melanie Taylor, Kingsley Agho

**Affiliations:** 1 Instituto Mexicano del Seguro Social, Delegación en Oaxaca, Mexico; 2 Asociación Oaxaqueña de Psicología A.C/Centro Regional de Investigación en Psicología, Oaxaca, Mexico; 3 Faculty of Medicine, Universidad Autónoma "Benito Juárez" de Oaxaca, Oaxaca, México; 4 School of Medicine, University of Western Sydney, Sydney, Australia

## Abstract

**Background:**

The A/H1N1 pandemic originated in Mexico in April 2009, amid high uncertainty, social and economic disruption, and media reports of panic. The aim of this research project was to evaluate the psychological response of family primary caregivers of patients hospitalised in the Intensive Care Unit (ICU) with suspected influenza A/H1N1 to establish whether there was empirical evidence of high adverse psychological response, and to identify risk factors for such a response. If such evidence was found, a secondary aim was to develop a specific early intervention of psychological support for these individuals, to reduce distress and possibly lessen the likelihood of post-traumatic stress disorder (PTSD) in the longer term.

**Methods:**

Psychological assessment questionnaires were administered to the family primary caregivers of patients hospitalised in the ICU in the General Hospital of Zone 1 of the Mexican Institute for Social Security (IMSS), Oaxaca, Mexico with suspected influenza A/H1N1, during the month of November 2009. The main outcome measures were ratings of reported perceived stress (PSS-10), depression (CES-D), and death anxiety (DAQ). Data were subjected to simple and multiple linear regression analysis to identify risk factors for adverse psychological response.

**Results:**

Elevated levels of perceived stress and depression, compared to population normative data, and moderate levels of death anxiety were noted. Levels of depression were similar to those found in comparable studies of family members of ICU patients admitted for other conditions. Multiple regression analysis indicated that increasing age and non-spousal family relationship were significantly associated with depression and perceived stress. Female gender, increasing age, and higher levels of education were significantly associated with high death anxiety. Comparisons with data collected in previous studies in the same hospital ICU with groups affected by a range of other medical conditions indicated that the psychological response reported in this study was generally lower.

**Conclusions:**

Data indicated that, contrary to widely publicised reports of 'panic' surrounding A/H1N1, that some of those most directly affected did not report excessive psychological responses; however, we concluded that there was sufficient evidence to support provision of limited psychological support to family caregivers.

## Background

A novel influenza of swine origin was first detected in Mexico during March and early April 2009 as increasing incidence of atypical respiratory disease in localised areas in Mexico was reported. Details of the epidemiology, spread, and risk factors for infection and death have been reported for early spread of the disease in Mexico [1,2].

Although initially thought to be the result of an extended seasonal influenza outbreak, the high level of hospitalisation and severe cases of pneumonia in young and otherwise healthy adults was unusual. In Oaxaca on 15 April 2009 health officials were notified of a suspected case of atypical pneumonia; the patient died within a few days. Investigation of this case identified a novel agent, later identified as a non-typeable strain of influenza A. On 23 April the Public Health Agency of Canada and the Communicable Diseases Center (CDC) in Atlanta confirmed that a common novel influenza A virus had been detected in two Mexican samples; the one from Oaxaca, and another from La Gloria, Veracruz, and was similar to a strain isolated from patients in California [3]. A week later on 29 April the World Health Organisation (WHO) announced a global pandemic alert level Phase 5 [4], indicating sustained human-to-human transmission in one WHO region of the world, and this was later raised to a global Phase 6 pandemic on 11 June 2011 [5], which was the pandemic alert level at the time of our study.

With uncertainty regarding the virulence and transmissibility of the pandemic in the early stages, and immense media scrutiny and reporting, there was widespread public fear [6]; and media reports of panic, especially in Mexico [7]. Even the most trusted source of global health information; WHO, was being reported in the media as warning that "all of humanity is under threat" [8]. High levels of fear and concern persisted in Mexico due to concerns about the severity of the illness, uncertainty surrounding its mortality rate, the susceptibility of younger and healthy people, and potential for contagion and stigma.

### Pandemic context at the time of this study

Our study was conducted from 1 to 30 November 2009. On 21 September 2009 the Government of Mexico announced that the country was at an "intermediate warning" level for influenza caused by A/H1N1 indicating that people should strengthen measures to promote health [9]. During the period from the start of the outbreak until 19 September, 220 people had died and 26,865 had been infected throughout the country, and 3,486 people had died and 296,000 had been infected, globally. The Health Secretary, José Ángel Córdova, reported that infection levels had accelerated in the States of Nuevo León, Baja California, Sinaloa in Mexico City, Tlaxcala and Oaxaca. At the time of the study, Mexico had experienced three peaks in infection rates; the first from 23 to 30 April, the second between 26 June and 24 July, and the third in mid September.

### Background research

Research in the area of psychological response of family members of patients has generally focused on the psychological assessment of family members or informal caregivers of patients admitted to ICUs [10], and has included assessments of post traumatic stress symptoms [11], psychological impacts of being involved in making end-of-life decisions and interventions to support families [12], and assessment of psychological or physical health of caregivers of patients on prolonged mechanical ventilation and the chronically critically ill [13,14]. Another source of research literature on the psychological response of caregivers has focused on the longer term mental and physical health burden on caregivers providing care for patients with long term conditions, such as HIV/AIDS, cancer, or dementia [15]. Therefore studies of caregiver psychological response are highly varied, both in terms of psychosocial impacts specific to different types of conditions (e.g. acute trauma and possible situations surrounding that, or fatal illness), their temporal features and outcomes with regard to care-giving (long-term care and eventual death, potential for recovery), and the psychological assessments used. In addition, there are differences in the time frames in which psychological symptoms are assessed in such studies; typically ranging from hospitalisation to 6-9 months post-discharge for ICU-related studies. However, one common aspect of most ICU studies of this nature is that they usually assess one or more of the following; stress, depression, and/or anxiety.

A recent review of symptoms experienced by family members of patients in ICUs [10] identified common risk factors for stress, anxiety and depression from 18 core studies. In terms of demographic risk factors, being female was a risk factor for most types of stress (including acute stress disorder and post-traumatic stress disorder (PTSD)) and depression, and being a spouse was a common risk factor for depression and anxiety.

Azoulay et al [11], in conclusion of their study of PTSD in family members of ICU patients, commented on the high levels of PTSD and the need for preventative and early intervention strategies. They suggest that high rates of anxiety and depression in family members may increase the risk of PTSD reaction and call for a need to identify factors detectable at the time of the ICU stay and associated with increased vulnerability in family members.

In our study, our focus was to evaluate levels of perceived stress, depression and death anxiety in the primary family caregiver of patients hospitalised and admitted to the ICU with suspected A/H1N1. Our aim was to empirically document the nature of the psychological impact of this epidemic in Oaxaca, to screen the primary family caregiver for adverse psychological symptoms, and to analyse data to identify risk factors for these adverse reactions. In addition, if evidence of adverse psychological response was found, we sought to develop appropriate resources to assist this population to cope with and reduce such responses, and in doing so, possibly lower levels of acute stress and likelihood of development of PTSD.

In this article we will report an overview of the levels of psychological response reported during the screening of these family caregivers; identify risk factors that are associated with an elevated adverse response; and reference the extent of this response by comparing our findings to comparable data collected by the research team at the same hospital, and from comparable ICU studies reported in the literature, as well as established normative population and community data for our psychological assessment tools. We will then provide a brief overview of the early psychological support intervention offered to family caregivers.

## Methods

### Participant selection

The research team assessed the psychological response of the family primary caregiver of all patients admitted to hospital, by ambulance, with respiratory distress and hospitalised in the ICU with suspected influenza A/H1N1 in the General Hospital of Zone 1 (HGZ 1) of the Mexican Institute for Social Security (IMSS) in Oaxaca, during 1-30 November 2009. Due to the infectious nature of the medical condition determining ICU admission and the need for stringent infection control only one relative of the patient is authorised to have contact with the patient in ICU. This primary caregiver is allowed access to communicate with the patient and to attend to their personal care. The authorised caregiver was the one approached to take part in the study.

Participation in the study was voluntary and anonymous. The only exclusion criterion for participation was a prior psychiatric diagnosis. The study was approved by the Research and Ethics Committee of the HGZ 1 of the IMSS and all participants provided written informed consent.

### Materials

The psychological response of the family primary caregiver was assessed using three established assessment tools:

- *Perceived Stress Scale (PSS-10) *[16]. The PSS-10 is a 10-item self report scale used to measure global perceived stress, it has been found useful as a predictor of physical symptoms and health outcomes. The scale assesses the respondent's appraisal of his/her life as unpredictable, uncontrollable, and overloaded during the preceding month. Scores range from 0 to 40 with higher scores indicating a higher risk factor for future distress.

- *Center for Epidemiologic Studies Depression Scale (CES-D) *[17]. The CES-D is a 20-item self-report scale developed for the general population to measure the frequency of depressive symptoms during the past week. It is not a clinical diagnostic tool, but has been used widely as a useful screening tool. It has excellent reliability (α coefficients, 0.85-0.91) and validity. Responses are rated on a four-point scale to yield total scores in the range 0 to 60. Higher scores indicate a greater risk of depression, with scores ≥16 indicating an increased risk of clinical depression and, possibly, mortality [18].

- *Death Anxiety Questionnaire (DAQ) *[19]. The DAQ is a 15-item self-report scale that measures attitudes towards one's own death and dying, including fear of the unknown, fear of suffering, fear of loneliness and fear of personal extinction. Death anxiety can be interpreted as an additional form of general anxiety or distress in the context of our study. Death anxiety has been linked to self-esteem and well-being, personality, valuing life, cultural values and differences and religiosity [20].

These scales were chosen for a range of reasons; the CES-D had been used in other clinical studies in ICUs to assess responses of caregivers and others [10], there were established normative data from populations and community based samples for all scales [17,19,21], and these scales had been used successfully in studies of the psychological impacts of a range of other medical conditions and situations previously conducted by the research team, allowing for direct comparisons to be made to these data [22].

### Procedure

A single interviewer collected data from all participants, in the period shortly after the patient was diagnosed and hospitalised in the hospital ICU. Participants were presented with each question and set of response options by the interviewer, and the interviewer noted each response and subsequently scored the data for each participant. Demographic data were also collected; age, gender, family relationship to patient and education level.

### Statistical analysis

Exploratory data analysis was conducted using frequency distribution for categorical variables and graphs and summary statistics for continuous variables. Continuous data for the psychological response scale variables were examined using regression analysis and checked for homogeneity of variance. Skewed distributions were natural logarithm transformed before simple and multiple linear regression analysis was performed. Statistical analyses were undertaken using the statistical package STATA, version 10 (2008; Stata Corporation, College Station, TX, USA). Statistical significance was taken as p ≤ 0.05.

## Results

### Characteristics of the sample

During the study period 36 patients were hospitalised and admitted to the ICU, and all were subsequently confirmed as having A/H1N1. Only one family member in the role of primary caregiver refused to participate in the study, and no family members in the primary caregiver role had a prior psychiatric diagnosis. Therefore the final study sample size was 35. Table 1 summarises the characteristics of the sample.

**Table 1:** Sample characteristics (n = 35)

Variables	
**Gender**	

Male	25.7%

Female	74.3%

**Age**	

Age (mean years ± SD)	(32 ± 7.3)

**Educational level**	

High school level or below	65.7%

University level	34.3%

**Family relationship**	

Spousal (husband/wife/partner)	42.9%

Non-spousal (mother/daughter/sibling)	57.1%

**Psychological response**	

Perceived Stress Scale (PSS-10) (mean ± SD)	(16.7 ± 7.9)

Depression Scale (CES-D) (mean ± SD)	(16.4 ± 5.8)

Death Anxiety (mean ± SD)	(15.1 ± 5.4)

Three quarter of the study participants were female (74.3%), 43% were in a spousal relationship with the admitted patient, and around a third (34.3%) had a university-level education. The mean age of participants was 32 (range 20-55).

**Figure 1:**
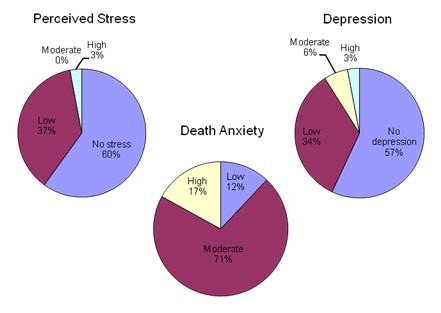
**Summary of psychological response assessments (n = 35)**.

The mean scores for perceived stress, depression, and death anxiety were 16.7, 16.4 and 15.1, respectively. These data were categorised, using established cut-off scores, and are shown in Figure 1 for the three assessment scales.

From Figure 1 it can be seen that the majority of participants reported no stress or depression (60% and 57%, respectively) and around a third of participants' responses were categorised as 'low' for stress and depression (37% and 34%, respectively). High levels of stress and depression were noted for a small proportion of participants (3% for both measures). Although the term 'low' has been used for categorisation of depression it should be noted that this represents the cut-off score of 16, above which individuals are regarded as being at higher risk of clinical depression i.e. 43% of the sample had a score above this cut-off. High levels of death anxiety were reported by 17% of participants, with the majority reporting moderate levels of death anxiety.

#### Regression Analyses

Univariate analysis, conducted using the continuous psychological response data, identified that the following were significantly associated with perceived stress (coefficient, R^2 ^and p-value): female gender (16.4, 0.23, 0.003), non-spousal family relationship (0.48, 0.83, < 0.001), and increasing age in years (17.6, 0.59, < 0.001). Simple regression analysis also indicated that the following were significantly associated with depression (coefficient, R^2^, p-value): female gender (2.5, 0.22, 0.004), non-spousal family relationship (2.60, 0.55, < 0.001), increasing age in years (0.08, 0.85, < 0.001), and university-level education (2.65, 0.34, < 0.001); and for death anxiety: female gender (16.1, 0.26, < 0.001), non-spousal family relationship (15.9, 0.57, < 0.001), increasing age in years (0.44, 0.83, < 0.001), and university-level education (15.8, 0.34, < 0.001). These results are summarised in Table 2.

**Table 2:** Simple regression analysis for psychological assessment scale data (coefficient, standard error, 95% confidence intervals, R2, and level of statistical significance)

Variables	Coefficient (SE)	95% CI	R^2 ^(p-value)
**Perceived Stress Scale (PSS-10)**			
**Gender**			
Male	0.00		
Female	16.44 (5.15)	(6.0, 26.91)	0.23 (0.003)
**Age**			
Age in years	0.48 (0.04)	(0.40, 0.56)	0.83 (< 0.001)
**Educational level**			
High School or below	0.00		
University	17.67 (4.08)	(9.37, 26.0)	0.35 (< 0.001)
**Family relationship**			
Spousal	0.00		
Non-spousal	17.6 (2.53)	(12.50, 22.74)	0.59 (< 0.001)
**Depression Scale (CES-D) (natural logarithm transformed)**			
**Gender**			
Male	0.00		
Female	2.5 (0.79)	(0.86, 4.09)	0.22 (0.004)
**Age**			
Age in years	0.08 (0.01)	(0.06, 0.09)	0.85 (< 0.001)
**Educational level**			
High School or below	0.00		
University	2.65 (0.63)	(1.36, 3.94)	0.34 (< 0.001)
**Family relationship**			
Spousal	0.00		
Non-spousal	2.60 (0.41)	(1.78, 3.43)	0.55 (< 0.001)
**Death Anxiety**			
**Gender**			
Male	0.00		
Female	16.1 (4.64)	(6.68, 25.54)	0.26 (0.001)
**Age**			
Age in years	0.44 (0.03)	(0.38, 0.51)	0.83 (< 0.001)
**Educational level**			
High School or below	0.00		
University	15.83 (3.81)	(8.10, 23.57)	0.34 (< 0.001)
**Family relationship**			
Spousal	0.00		
Non-spousal	15.95 (2.34)	(11.12, 20.78)	0.57 (< 0.001)

SE = Standard Error; CI = Confidence Interval

Multivariate analysis, summarised in Table 3, indicated that the following were significantly associated with perceived stress (coefficient; 95% CI, p-value): increasing age in years (0.34; 0.24-0.44, < 0.001) and non-spousal relationship (6.79; 2.81-10.77, 0.002); depression (coefficient; 95% CI, p-value): increasing age in years (0.05; 0.04-0.07, < 0.001), non-spousal relationship (0.80; 0.20-1.39, 0.010) and female gender (4.85; 0.47-9.22, 0.031); and death anxiety: increasing age in years (0.32, 0.23-0.41, < 0.001), and university-level education (5.99, 2.44-9.54, 0.002).

**Table 3:** Multivariate regression analysis for psychological assessment scale data (coefficient, standard error, 95% confidence intervals, level of statistical significance, and R2)

Variables	Coefficient (SE)	95% CI	p-value
**Perceived Stress Scale (PSS-10)**			
**Gender**			
Male	0.00	-	-
Female	3.87 (2.40)	(-1.03,8.77)	0.117
**Age**			
Age in years	0.34 (0.05)	(0.24,0.44)	< 0.001
**Educational level**			
High School or below	0.00	-	-
University	1.04 (2.31)	(-3.66,5.74)	0.655
**Family relationship**			
Spousal	0.00	-	-
Non-spousal	6.79 (1.95)	(2.81,10.77)	0.002
	(R^2 ^= 0.88, p-value < 0.001)		
**Depression Scale (CES-D) (natural logarithm transformed)**			
**Gender**			
Male	0.00	-	-
Female	0.46 (0.36)	(-0.26,1.19)	0.204
**Age**			
Age in years	0.05 (0.01)	(0.04,0.07)	< 0.001
**Educational level**			
High School or below	0.00	-	-
University	0.09 (0.34)	(-0.61,0.79)	0.805
**Family relationship**			
Spousal	0.00	-	-
Non-spousal	0.80 (0.29)	(0.20,1.39)	0.010
	(R^2 ^= 0.89, p-value < 0.001)		
**Death Anxiety**			
**Gender**			
Male	0.00	-	-
Female	4.85 (2.14)	(0.47,9.22)	0.031
**Age**			
Age in years	0.32 (0.04)	(0.23,0.41)	< 0.001
**Educational level**			
High School or below	0.00	-	-
University	5.99 (1.74)	(2.44,9.54)	0.002
**Family relationship**			
Spousal	0.00	-	-
Non-spousal	0.10 (2.06)	(-4.10,4.29)	0.962
	(R^2 ^= 0.89, p-value < 0.001)		

SE = Standard Error; CI = Confidence Interval

### Comparison data

The research team has used the same methodology and assessment measures in small studies, also at the Oaxaca General Hospital, evaluating the psychological responses of relatives and patients to three other medical conditions or situations, those being: relatives of patients admitted to the Intensive Care Unit (ICU) (Vargas-Mendoza & Aguilar, unpublished data), patients who encountered foetal death (Vargas-Mendoza & Pacheco-Chávez, unpublished data), and patients undergoing haemodialysis in ambulatory care [22]. A further aim of the current study was to compare the psychological response to A/H1N1 with data gathered in these other studies. Categorical data from these studies have been summarised, alongside findings from the current study, in Table 4. Chi square statistical tests (Fishers exact), have been used to test for statistically significance differences.

**Table 4:** Summary of comparison data from studies undertaken at the Oaxaca General Hospital, using some of the same assessment tools

Psychological response		Influenza A/H1N1 (n = 35^a^)	Intensive Care Unit (n = 20^a^)	Foetal death (n = 10^b^)	Haemo-dialysis (n = 10^b^)	Chi-square p-value
**Perceived Stress (PSS-10)**	No stress	21/35 (60)	2/20 (10)	-	-	< 0.000
	Low	13/35 (37)	10/20 (50)	-	-	
	Moderate	0/35 (0)	6/20 (30)	-	-	
	High	1/35 (3)	2/20 (10)	-	-	
**Depression (CES-D)**	No depression	20/35 (57)	-	3/10 (30)	-	0.111
	Low	12/35 (34)	-	4/10 (40)	-	
	Moderate	2/35 (6)	-	3/10 (30)	-	
	High	1/35 (3)	-	0/10 (0)	-	
**Death Anxiety (DAQ)**	Low	4/35 (12)	-	-	3/10 (30)	0.327
	Moderate	25/35 (71)	-	-	5/10 (50)	
	High	6/35 (17)	-	-	2/10 (20)	

Proportions are shown with percentage in parenthesis. ^a ^relatives of the patient, ^b ^patients.

Comparisons with similar studies conducted at the Oaxaca General Hospital indicated that there was a statistical difference between the levels of perceived stress of family members of patients admitted to the ICU for A/H1N1 and for other reasons. Comparing the pattern of response it appears that the perceived stress levels in relation to A/H1N1 were lower. There did not appear to be statistical differences between the current A/H1N1 study data and equivalent data collected for other medical conditions in relation to depression or death anxiety.

## Discussion

This small study has provided evidence of a moderate psychological response in the family members of patients hospitalised in the ICU for A/H1N1. In the context of a novel influenza pandemic we have not found evidence that the level of response has been as excessive or alarming, as might have been predicted from reports in the media, and we found no evidence of panic or an 'epidemic of panic'.

We note that the majority of family members reported sub-threshold levels of stress and depression (60% and 57%, respectively); however 43% of participants reported levels of depression above the established cut-off score for higher risk of clinical depression. This was higher than levels recorded for caregivers of patients who had been mechanically ventilated for > 48 hours in ICU (30% at 2 months) [13] but much lower than caregivers of chronically critically ill patients when in ICU (75% at ICU enrolment) and similar to levels at 2 months post discharge (43%) [14]. In their review of ICU studies, McAdam and Puntillo [10] conclude that depression affected 15%-35% of patient family members, however, they too are comparing studies with inconsistent time frames used to examine symptoms, different medical conditions, and assessment instruments.

With regard to reported levels of stress, the PSS-10 has not been used by researchers evaluating stress in family members of patients in ICUs or generally, and therefore, this comparison is not available. In our study the mean stress score for the sample was equivalent to that recorded in a large heterogeneous European Spanish "stressed" sample of people coping with a range of adversities [23], suggesting that our sample could also be regarded as 'stressed'. Reported normative data for PSS-10 for normal healthy adults range from mean scores of 14.2 (SD 6.2) for those aged 18-29 years, up to 11.9 (SD 6.9) for those aged 55-64 years [21]. Our study sample mean of 16.7 (SD 7.9) would appear to be elevated compared to healthy norms.

Levels of death anxiety were generally much higher (often double) mean scores reported for heterogeneous groups [19], with just under three quarters of family members (71%) reporting moderate levels of death anxiety and 15% reporting high death anxiety. Given that all patients in this study had been admitted to the hospital ICU via ambulance in a state of respiratory distress it is possibly understandable that this would stimulate thoughts of potential death of the patient and bring feelings of one's own mortality into consciousness.

Simple univariate statistical analysis has shown associations between heightened psychological response in family members who are female, older, with higher levels of education and who are in a non-spousal relationship with the admitted patient. However, when subjected to multivariate analysis the most consistent association, across all measures, was an increased psychological response with increasing age. The reason for this finding is unclear. In general, caregiver age has not been reported as a significant risk factor in studies of stress and depression in caregivers in acute clinical settings [10]. It is likely that older participants are more likely to be older than the patient (the maximum age in our sample was 55), so one possible explanation is that the emotional response to the potential loss of someone younger may be more acute. The finding that increasing age is associated with increasing death anxiety is also an interesting finding. The generally accepted relationship between age and death anxiety is that death anxiety decreases across life span, although there is evidence that this decrease occurs from midlife, i.e. beyond the age of the majority of our study sample [24], and the impact of sudden mortality salience on death anxiety does not appear to be studied.

In addition to the effect of increasing age in the multivariate analysis, higher stress response and depression was noted for those in non-spousal relationships with the patient, i.e. in our sample these were mothers, daughters, brothers and sisters. This finding differs from other studies of family members of patients in ICUs where family relationship has been found to be a risk factor for adverse psychological response; in those studies spousal relationship has been found to be associated with higher depression [10]. It is interesting to note that family relationship was found not to be associated with death anxiety in the multivariate analysis. Here, higher levels of education and being female were the factors most strongly associated with higher reported death anxiety. Higher death anxiety is generally noted in females [20].

Comparing our current study data with similar prior studies conducted at the Oaxaca General Hospital it was interesting to note that, with a degree of confidence, the levels of stress reported in family members in response to patients admitted to the ICU with suspected A/H1N1 was lower than equivalent data reported by family members of patients admitted to the ICU for other medical conditions. Although one needs to be cautious when interpreting data based on small samples, this finding does add support to a lack of evidence of an extreme psychological response or 'panic' in association with pandemic A/H1N1, in the country most severely impacted.

### Limitations and strengths

This study has a number of limitations that need consideration. Firstly, it is based on a small sample of primary family caregivers and therefore the findings can only be regarded as indicative. Also the psychological assessments undertaken provide a single snapshot of how family caregivers were feeling close to the time of admission of the patient to the ICU, and do not therefore provide an indication of longer term psychological trajectories. There was also no opportunity to control for extraneous factors, that may have influenced caregivers' psychological condition, e.g. other life events, physical health status, and therefore it is not possible to identify psychological response attributable to the patient's condition and to pandemic A/H1N1 per se. Despite these limitations, participants did not have a history of psychiatric illness, and with regard to the main aim of the study; which was to identify if there was evidence for an adverse psychological response in family caregivers of patients admitted to ICU for A/H1N1 to support provision of psychological support, the evaluation that was undertaken adequately suited this purpose.

### Clinical Outcome

In reviewing data from our study we believed that there was evidence of moderate psychological response and that this confirmed the need for a level of psychological support to the families of patients hospitalised for A/H1N1. Therefore, in response we developed a psychological support strategy based on four principles, as follows:

1. *Provide supportive information*. The threat of pandemic influenza for our patients and their families can be a stressful event. It is important that they receive timely and adequate information concerning how to take care of, and protect, their loved ones. Such information increases a sense of control and self-efficacy and enables them to respond and support their loved one and other family members at this difficult time.

2. *Acknowledge their psychological response*. In addition, we need to let family members know that if someone close to them is sick it is normal to have a range of feelings such as feeling concerned by newscasts and media reports; feeling anxious, irritable or impatient; or losing the ability to concentrate on tasks.

3. *Confront stress*. Advise families to continue with normal life, to take time to eat, exercise, and rest, and to keep busy and focus on daily activities. Avoid drugs and alcohol. Stay in touch with friends and family and pay attention to television and radio reports that provide information on how to stay healthy and safe. Encourage them to talk to someone about their feelings if they are fearful or concerned.

4. *Consider the response of children: *To help children we advise that family members express what they feel and explain that people may feel concerned and that it is normal when they have stress. Give them information they can understand. Tell them that you will protect them so that they feel reassured. Encourage them to make drawings and paintings. These projects help to express what they feel. Touch and embrace them frequently. Keep to your routines with laughter and games. Teach them protective behaviours to protect them of infectious diseases; such as washing hands.

## Conclusion

This study sought to evaluate the psychological response of family primary caregivers of patients hospitalised in the ICU for suspected influenza A/H1N1 to establish whether there was evidence of an adverse psychological response, to identify risk factors for such a response, and to assess if the level of response was sufficient to support development of a specific package of psychological support for these individuals. Our data provided evidence of elevated perceived stress, depression, and death anxiety, particularly in caregivers who were older, or female, or in non-spousal relationships with the patient, and were in excess of levels that would have been predicted from normative population data and were generally comparable, or slightly lower, that levels reported elsewhere in ICU caregiver studies. Consequently we have developed a simple low level psychological support intervention as a form of psychological first aid to reduce acute stress and other adverse psychological reactions in these caregivers, and hopefully to reduce the likelihood of the development of PTSD.

## Key Messages

▪ When screened shortly after patient admission to ICU, family caregivers of patients with suspected A/H1N1 reported moderately elevated levels of stress and depression and high levels of death anxiety.

▪ Comparisons with published ICU studies and additional data from the same hospital suggested that caregivers of ICU patients with suspected A/H1N1 did not report higher levels of adverse psychological response than caregivers of patients admitted for other medical reasons.

▪ Older caregivers and those in non-spousal relationships with the patient were at higher risk of elevated stress and depression.

▪ Data supported the need for some low level psychological support for caregivers of A/H1N1 patients in the ICU.

▪ Even though this sample was highly A/H1N1 pandemic-affected, there was no evidence to support the media image of a panic-stricken public.

## List of Abbreviations

A/H1N1: Influenza A, variant H1N1 the pandemic strain of influenza; CES-D: Center for Epidemiologic Studies Depression Scale; DAQ: Death anxiety questionnaire; HGZ1: General hospital Zone 1; ICU: Intensive Care Unit; IMSS: Mexican Institute for Social Security; PSS-10: Perceived Stress Scale (10 item); PTSD: post-traumatic stress disorder.

## Competing interests

The authors declare that they have no competing interests.

## Authors' contributions

JE-R and JEV-M conceived the study. All authors were involved in study development under the leadership of JE-R. JEV-M supervised the data collection and psychological assessment, MM-G, CM-Z helped with caring for the families and patients and supervised AE-C who conducted the interviews and initial data analysis, JE-R and JEV-M drafted the first manuscript and translated it into English, MT developed the draft and final version of the manuscript, assisted with analysis and interpretation of the data, and is the corresponding author, KA conducted the statistical analysis, and contributed to the data interpretation and draft manuscript. All authors reviewed the final version of the manuscript.

## Authors' Information

JE-R is Coordinator of Health Research in the IMSS and is Professor Investigator in the Faculty of Medicine at the Benito Juarez University of Oaxaca. His area of research is mental health. JEV-M is a Clinical psychologist and Chief of Psychology Services at IMSS, Honorary President of Oaxaqueña Association of Psychology, and is interested in the mental health implications of medical conditions. MM-G and CM-Z are internists and doctors of internal medicine services and AE-C is an MD with interest in health research; all are at IMSS and are in the Faculty of Medicine at the Benito Juarez University of Oaxaca. MT and KA are researchers in the Disaster Response and Resilience Research Group of the School of Medicine at the University of Western Sydney; they are working on population threat perception to pandemic and the psychosocial impacts of emergency disease outbreaks in humans and animals.

## Pre-publication history

The pre-publication history for this paper can be accessed here:

http://www.biomedcentral.com/1471-244X/10/104/prepub
